# Menor Prevalência e Extensão da Aterosclerose Coronária na Doença de Chagas Crônica por Angiotomografia Coronária

**DOI:** 10.36660/abc.20200342

**Published:** 2020-12-01

**Authors:** Savio Cardoso, Clerio Francisco de Azevedo, Fábio Fernandes, Barbara Ianni, Jorge Andion Torreão, Mateus Diniz Marques, Luiz Francisco Rodrigues de Ávila, Raul Santos, Charles Mady, Roberto Kalil-Filho, José Antônio Franchine Ramires, Marcio Sommer Bittencourt, Carlos E. Rochitte

**Affiliations:** 1 Universidade de São Paulo Faculdade de Medicina Hospital das Clínicas São PauloSP Brasil Universidade de São Paulo Faculdade de Medicina Hospital das Clínicas Instituto do Coração, São Paulo, SP - Brasil; 2 Duke University Hospital - Medicine/Cardiology North Carolina EUA Duke University Hospital - Medicine/Cardiology, North Carolina - EUA; 3 Hospital Universitário de Santa Maria Santa MariaRS Brasil Hospital Universitário de Santa Maria, Santa Maria, RS - Brasil; 4 Hospital Sírio-Libanês São PauloSP Brasil Hospital Sírio-Libanês, São Paulo, SP - Brasil; 5 Hospital Israelita Albert Einstein São PauloSP Brasil Hospital Israelita Albert Einstein, São Paulo, SP - Brasil; 6 Universidade de São Paulo Hospital Universitário de São Paulo São PauloSP Brasil Universidade de São Paulo Hospital Universitário de São Paulo, São Paulo, SP - Brasil; 7 Hospital do Coração Associação Beneficente Síria São PauloSP Brasil Hospital do Coração (HCOR) Associação Beneficente Síria, São Paulo, SP - Brasil

**Keywords:** Doença de Chagas/fisiopatologia, Aterosclerose, Doença Arterial Coronariana, Tomografia Computadorizada/métodos, Escore de Cálcio

## Abstract

**Fundamento:**

Em regiões endêmicas da doença de Chagas, por muitos anos, existe uma observação empírica recorrente de que a doença arterial coronariana (DAC) é incomum em pacientes com doença de Chagas. Estudos anteriores baseados em análise patológica ou angiografia coronária invasiva apresentam resultados controversos.

**Objetivo:**

Investigar se a DAC é menos prevalente e menos grave em pacientes com doença de Chagas crônica em comparação a uma população pareada controle, com perfil de risco para DAC similar.

**Métodos:**

Um total de 86 participantes, 43 pacientes com doença de Chagas crônica consecutivos e 43 indivíduos assintomáticos, sem qualquer história prévia de doença cardíaca ou doença DAC conhecida (grupo controle), foram incluídos no estudo. Pacientes e controles foram pareados quanto sexo, idade e escore de risco de Framingham. Todos os pacientes foram analisados quanto ao escore de cálcio coronário (ECC) e submetidos à angiotomografia coronária usando um tomógrafo de 320 detectores. O nível de significância estatística adotado foi de p < 0,05.

**Resultados:**

O ECC foi significativamente mais baixo em pacientes com doença de Chagas em comparação aos controles (p<0,05). A presença de placas ateroscleróticas coronárias foi significativamente menos frequente em pacientes com doença de Chagas que nos controles (20,9% versus 41,9%, p=0,037). Após ajuste quanto ao escore de Framingham, o odds ratio para a presença de qualquer calcificação coronária foi de 0,26 (IC95%: 0,07-0,99, p=0,048). O padrão é similar para escore de cálcio coronário (ECC) > 10 (OR: 0,11, IC95%: 0,01-0,87, p=0,04), e para a presença de estenose (OR: 0,06, IC95%: 0,01-0,47, p=0,001). O pareamento por escore de propensão também mostrou um efeito da doença de Chagas no ECC (-21,6 pontos no escore absoluto e 25% menos pacientes com ECC > 10; p=0,015).

**Conclusões:**

A prevalência e a gravidade da DAC são mais baixas nos pacientes com doença de Chagas crônica em comparação a uma população pareada e perfil de risco para DAC similar. (Arq Bras Cardiol. 2020; 115(6):1051-1060)

## Introdução

Aproximadamente 20 000 pessoas morrem anualmente de doença de Chagas e 100 milhões de pessoas estão em risco de contrair a infecção em todo o mundo. A doença é endêmica em grande parte do México, América Central, e América do Sul, onde cerca de seis milhões de pessoas são infectadas.^1 , 2^ Nos Estados Unidos, estima-se que 300 000 indivíduos estejam infectados com *Trypanosoma cruzi* ( *T. cruzi* ), a maioria dos quais adquiriram a infecção em áreas endêmicas e depois migraram para a América do Norte.^3 - 5^

A cardiomiopatia chagásica é essencialmente uma miocardite. O processo inflamatório, apesar de mais intenso na fase aguda, é clinicamente silencioso, mas incessante em pacientes com infecção crônica.^6 , 7^ Após a fase aguda da doença, os pacientes entram na fase indeterminada, a qual é assintomática e pode ter duração indefinida. Cerca de 20-30% das pessoas com infecção crônica por *T. cruzi* eventualmente desenvolvem doença clínica, predominantemente cardíaca. A doença cardíaca geralmente inicia-se com anormalidades de condução, tais como bloqueio de ramo direito e/ou bloqueio fascicular anterior esquerdo, que pode ser seguido por cardiomiopatia dilatada anos depois.^8^ A insuficiência cardíaca e outras anormalidades ^9 - 14^associadas, tais como disautonomia simpática,^15^ podem ser observadas nesta fase. Em estágios posteriores, aneurismas apicais e formação de trombos podem ser achados frequentes.

Durante a fase crônica da cardiomiopatia, uma proporção importante de pacientes queixa-se de dor torácica atípica. Apesar disso, a prevalência de doença arterial coronariana (DAC) nesses pacientes é consistentemente baixa. Portanto, especialistas em doença de Chagas levantaram a hipótese de que pacientes com doença de Chagas teriam menos DAC que indivíduos com perfil de risco similar para DAC. mas sem doença de Chagas. Ainda, a relação entre doença de Chagas e doença aterosclerótica coronariana permanece controversa.^16^ Foi demonstrado que uma perfusão miocárdica anormal e mesmo cicatriz miocárdica extensa podem ocorrer em pacientes chagásicos na ausência de estenose coronária epicárdica.^17 , 18^ A fibrose miocárdica em pacientes com doença de Chagas pode ser detectada em fases iniciais da doença e precisamente quantificada por ressonância magnética cardíaca,^19 , 20^ e sua magnitude tem significância prognóstica comprovada.^21^

Apesar da alta prevalência de fatores de risco para DAC, incluindo tabagismo, estudos iniciais que indicavam uma baixa prevalência de DAC significativa por angiografia coronária invasiva em pacientes com cardiomiopatia chagásica grave sugeriram uma prevalência distinta de DAC em pacientes com doença de Chagas.^22 , 23^ A influência da doença de Chagas sobre a DAC e outras doenças crônicas degenerativas, incluindo doenças neoplásicas,^24^ tem plausibilidade biológica baseada na presença de fatores e mecanismos comuns, tais como inflamação crônica, fibrose, aumento de níveis de radicais livres e de lipoproteína de baixa densidade, e diminuição nos níveis de óxido nítrico. No entanto, estudos que descreveram a prevalência de DAC em pacientes com doença de Chagas relataram resultados conflitantes. Enquanto alguns estudos encontraram uma prevalência similar à observada na população geral,^23^ vários estudos sugeriram que a prevalência de DAC em pacientes com doença de Chagas poderia ser menor que àquela encontrada na população não infectada com escores de clínicos de risco similares para DAC.^17 , 22 , 25 - 27^ Também existe evidência básica preliminar sugerindo que a infecção por *T. cruzi* , por si só, possa exercer um efeito protetor contra o desenvolvimento de DAC em indivíduos cronicamente infectados.^28^ Isso seria possível pela ação potencial da trans-sialidase produzida por *T. cruzi* em reduzir a infecção por micoplasma, o qual poderia estar envolvido na inflamação associada à DAC.^28^

No presente estudo, utilizamos técnicas modernas de tomografia computadorizada cardíaca para determinar se pacientes com doença de Chagas crônica apresentam uma prevalência e/ou gravidade mais baixa da DAC em comparação a uma população pareada, de indivíduos assintomáticos e sem história prévia de DAC, mas com perfil risco similar para DAC.

## Métodos

### População

Um total de 43 pacientes consecutivos com doença de Chagas crônica foram recrutados prospectivamente de nosso ambulatório especializado em doença de Chagas. Todos os pacientes apresentavam sorologia positiva para doença de Chagas por pelo menos duas técnicas diferentes: ELISA e imunofluorescência indireta. Os pacientes foram classificados em três subgrupos: (1) pacientes na fase indeterminada; (2) pacientes com anormalidades eletrocardiográficas, e (3) pacientes com insuficiência cardíaca. O subgrupo indeterminado (15 pacientes, 34,9%) foi definido como pacientes assintomáticos com doença de Chagas crônica, mas sem anormalidades no raio-X de tórax, eletrocardiograma (ECG), ecocardiografia ou estudos de raio-X contrastado do esôfago e do cólon. O grupo ECG (n=12, 27,9%) foi definido como pacientes com anormalidades no ECG, mas sem anormalidades na função sistólica do ventrículo esquerdo global ou segmentar, e com fração de ejeção do ventrículo esquerdo (FEVE) ≥ 55% pela ecocardiografia. Pacientes com FEVE < 55% foram alocados no subgrupo de cardiomiopatia (n=16, 37,2%).

Além disso, um total de 43 indivíduos assintomáticos, sem história de doença cardíaca ou DAC, e sem história familiar de DAC precoce, que foram submetidos à angiotomografia coronariana somente para estratificação de risco foram incluídos no grupo controle. Esses 43 pacientes foram selecionados de um grupo controle maior de 124 indivíduos consecutivos que preencheram os critérios de pareamento para cada paciente com doença de Chagas. Um segundo indivíduo que preencheu os critérios de pareamento não foi incluído, e foi alocado no grupo controle “backup”. Para a seleção do grupo controle, três níveis de paramento foram usados para cada paciente com doença de Chagas (caso/controle: 1x1): primeiro, um escore de risco de Framingham (FRS) dentro da mesma categoria de risco (baixo, intermediário e alto);^29^ segundo diferença de idade de até 5 anos; e terceiro, mesmo sexo. Ainda, uma vez que não havia pacientes com diabetes mellitus no grupo doença de Chagas, também excluímos pacientes diabéticos do grupo controle. O FRS foi calculado usando o modelo descrito por D’Agostino et al.,^16^ e se baseou nos seguintes parâmetros clínicos: idade, lipoproteína de alta densidade (HDL colesterol) e colesterol total, pressão arterial sistólica, tratamento para hipertensão, tabagismo e diabetes. Todos os 43 indivíduos foram submetidos a teste sorológico para excluir doença de Chagas.

Todos os pacientes assinaram um termo de consentimento aprovado pelo comitê de ética institucional local. Em ambos os grupos, nós excluímos pacientes com insuficiência renal, definida por creatinina sérica > 1,5mg/dL e/ou *clearance* de creatinina <60mL/min/1,72m^2^, pela fórmula MDRD ( *Modification of Diet for Renal Disease* ).

### Angiotomografia coronariana (ATC)

Todos os pacientes foram submetidos à ATC utilizando-se um tomógrafo de 320 detectores ( *AquillionOne* ^TM^ *– Canon Medical Systems Corporation,* Otawara, Japão) após jejum de pelo menos 4 horas. Antes do exame, os pacientes responderam um questionário sobre risco cardiovascular e foram avaliados com medidas de frequência cardíaca e pressão arterial.

O protocolo de aquisição incluiu o escore de cálcio coronário (ECC) e ATC. Para determinação do ECC, utilizou-se tempo de rotação do tubo de 370ms, voltagem do tubo de 120kV, corrente de 300mA, colimação 321x0,5mm com imagens reconstruídas com espessura de corte de 3mm, obtidas a cada batimento durante diástole.

Para a ATC, os participantes com frequência cardíaca acima de 65bpm receberam até 20mg de metoprolol endovenoso. Todos os participantes receberam nitrato sublingual se apresentassem pressão arterial sistólica maior que 90 mmHg. Os limites superior e inferior foram definidos na imagem do ECC. Um rastreamento em tempo real do contraste (método do “ *bolus tracking* ”) ( *Sure Start* ^TM^ – *Toshiba Medical Systems Corporation,* Otawara, Japão) foi usado para detectar aumentos acentuados na intensidade do sinal na aorta descendente. A aquisição iniciou-se no limiar de 150 HU. Um contraste iodado não iônico (70mL, concentração de iodo de 370mg/mL) (Iopamiron 370 – Schering, São Paulo, Brasil, sob licença da BRACCO – Itália), foi administrado por uma bomba injetora ( *Stellant* ^TM^ – *Medrad,* Indianola, PA, EUA) a uma taxa de 5mL/s seguido por 40mL de solução salina ( *saline chaser* ).

A aquisição de imagem por ATC ocorreu durante um único batimento (frequência cardíaca menor que 65bpm) de acordo com um protocolo de aquisição prospectiva por ECG e durante apneia inspiratória.^30 , 31^ Os parâmetros de aquisição da TCA dependeram do índice de massa corporal (IMC): até 23 kg/m^2^, voltagem do tubo de 100kV e corrente de 450-550mA; IMC de 24 a 39 kg/m^2^, 120kV e 400-580mA, respectivamente; para IMC maior que 40kg/m^2^, 135kV e 510mA, respectivamente. A colimação dependeu da dimensão longitudinal do coração e variou de 120 a 160mm, com aquisições de cortes de espessura de 0,5mm (240x0.5mm a 320x0.5mm). A dose de radiação típica para este protocolo é de até 10 mSv.

### Análise de ATC

A análise do ECC foi por meio do protocolo padrão descrito por Agatston et al.,^32^ utilizando-se no mínimo três pixels maiores ou iguais a 130HU para identificação de cálcio.

As imagens obtidas por ATC foram reconstruídas imediatamente após a conclusão do escaneamento de maneira consistente, para identificar imagens da artéria coronária livres de movimento. Os dados acoplados ao ECG foram reconstruídos a 75% do ciclo cardíaco. Em caso de qualidade insuficiente da imagem, foram reconstruídas fases adicionais com acréscimos de 5%. Múltiplas fases foram usadas para interpretação da imagem se a fase de mínima movimentação da artéria coronária tivesse sido diferente para artérias diferentes. Todas as imagens foram analisadas por dois especialistas em ATC que desconheciam qualquer informação clínica, usando a estação de trabalho ( *workstation* ) *Vitrea* ^TM^ *FX* ( *Vital Images Inc,* Plymouth, MN, EUA). Os avaliadores poderiam usar quaisquer ferramentas disponíveis na estação de trabalho para analisar as imagens, tais como cortes axiais, multiplanar e reformatação curva, projeção de intensidade máxima e reconstrução em 3D. Em caso de discrepâncias, um consenso entre os dois avaliados foi obtido quanto à presença e tipo de placa aterosclerótica e grau de estenose por cada segmento coronário. Para a segmentação coronária, usamos um modelo de 19 segmentos, previamente descrito no estudo CorE-64.^33^

**Figura 1:**
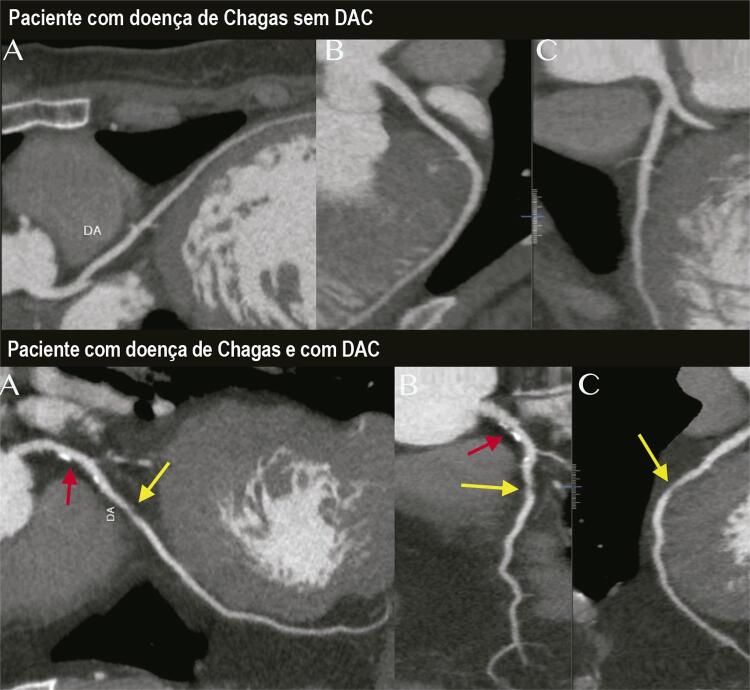
– Imagens de angiotomogafia coronariana de exemplos de casos de pacientes com doença de Chagas sem (linha superior) e com (linha inferior) doença arterial coronariana (DAC). Imagens A, B e C mostram três diferentes cortes da artéria descendente anterior (ADE) esquerda de ambos os pacientes. Na linha inferior, as setas vermelhas indicam placa parcialmente calcificada na ADE proximal sem estenose (placa mista predominantemente calcificada), e setas amarelas indicam uma placa não calcificada com estenose luminal importante.

A DAC foi definida como a presença de placa aterosclerótica coronariana, mesmo na ausência de obstrução luminal. DAC obstrutiva importante foi definida como redução do lúmen maior ou igual a 50% do diâmetro luminal de referência (segmento imediatamente distal sem evidência de doença). A placa aterosclerótica foi classificada por análise qualitativa: A – placa não calcificada, B – placa predominantemente não calcificada; C – placa mista, D – placa predominantemente calcificada e E – placa calcificada. Quando mais de um tipo de placa estava presente, considerou-se o tipo predominante em cada paciente para análise. O grau de estenose coronária foi visualmente classificado pelos dois avaliadores segundo o escore de estenose: 0 – ausência de redução luminal, 1-estenose leve (< 50%), 2- estenose moderada (50-69%) e 3 – estenose grave (≥ 70%). A Figura 1 mostra dois exemplos de pacientes com doença de Chagas com e sem DAC.

### Análise estatística

Todas as variáveis contínuas são apresentadas em média ± desvio padrão e todas as variáveis categóricas descritas como porcentagem ou número absoluto. As variáveis contínuas com distribuição normal foram descritas em média e desvio padrão, e as variáveis contínuas sem distribuição normal foram descritas em mediana e intervalo interquartil. O teste de Shapiro-Wilk foi usado para avaliar normalidade da distribuição. Para comparação entre grupos, usamos o teste t de Student para amostras pareadas para variáveis com distribuição normal, e o teste de Mann-Whitney para variáveis não paramétricas. Para comparações de variáveis categóricas, usamos o teste qui-quadrado de Pearson ou o teste exato de Fisher, conforme apropriado. Para a análise multivariada, realizamos a análise de regressão logística condicional ajustada para o FRS para cada um dos desfechos binários de ECC ≥0; ECC< 10 ou > 10 e presença de obstrução coronária (estenose >50% na avaliação visual). O pareamento por escore de propensão usando o método de kernel e reamostragem ( *bootstrapping* ) foi usado para pareamento adicional de pacientes com doença de Chagas e controles. Não existem dados na literatura sobre a prevalência da placa aterosclerótica coronária, medida por tomografia computadorizada (TC), em pacientes com doença de Chagas; por isso, usamos um tamanho amostral exploratório ou de conveniência. O estudo foi delineado para ter um grupo controle pareado, de maneira que 43 pacientes com doença de Chagas tinham que ser pareados a 43 controles sadios, com sexo, faixa etária, e FRS similares. As análises foram realizadas com o programa Stata, versão 13.0 (StataCorp, College Station, Texas). Todos os testes foram bicaudais, e um valor de p<0,05 foi considerado como indicativo de significância estatística.

## Resultados

As características clínicas dos participantes são apresentadas na Tabela 1 . No grupo de pacientes com doença de Chagas, 27 pacientes eram mulheres (62,8%) e a idade média era de 54,2±8,3 anos. No grupo controle, 27 pacientes eram mulheres (62,8%) e a idade média era de 55,0±7,1 anos. Idade, sexo e FRS foram similares, destacando o pareamento efetivo de ambos os grupos. Os pacientes com doença de Chagas apresentavam níveis mais elevados de HDL colesterol que o grupo controle (p=0,030, Tabela 1 ). Nenhum paciente foi excluído devido à baixa qualidade de imagem da TCA, e não foi relatado nenhum evento adverso relacionado aos procedimentos do estudo.

**Tabela 1:** – Características clínicas

Características	Grupos		p
	Doença de Chagas (n=43)	Controle (n=43)	
**Idade (anos)**	**54,18 ± 8,26**	**55,00 ± 7,12**	0,626*
Sexo masculino	16 (37,2%)	16 (37,2%)	1,000**
Índice de Massa Corporal	27,4±4,2	28,9±4,8	0,127*
FC (bpm) (ATC)	63,4±5,1	62,2±3,7	0,215*
HDL colesterol (mg/dL)	51,0 ± 12,4	45,9 ± 9,2	0,030*
Total Colesterol (mg/dL)	201,5 ± 36,3	224,3 ± 84,6	0,108*
Hipertensão tratada	20 (46,5%)	24 (55,8%)	0,388**
Tabagismo atual	04 (9,3%)	04 (9,3%)	1,000**
Escore de risco de Framingham	3 (1; 8)^†^	4 (1; 8)^†^	0,682***

FC: frequência cardíaca; ATC – angiotomografia coronária; HDL: lipoproteína de alta densidade; bpm: batimentos por minuto. †: Mediana (P25; P75); *: Teste t de Student; **: Teste qui-quadrado de Pearson; ***: Teste de Mann-Whitney .

**Figura 2:**
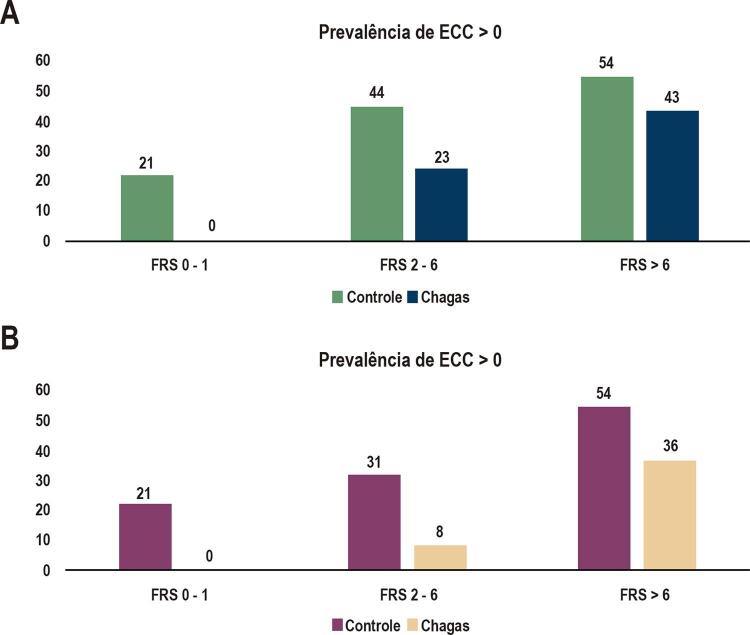
– Relação entre doença de Chagas e prevalência de doença arterial coronariana (DAC). A. Prevalência de qualquer calcificação coronariana (escore de cálcio coronário, ECC, > 0) em pacientes com doença de Chagas e controles, estratificados pelos tercis de escore de risco de Framingham (FRS). B. Prevalência de ECC > 10 em pacientes com doença de Chagas e controles, estratificados pelos tercis de escore de risco de Framingham.

O ECC foi significativamente mais baixo nos pacientes com doença de Chagas que nos controles. Ainda, a gravidade da estenose coronária, número de territórios de perfusão coronária, e número de segmentos coronários com DAC foi significativamente menor no grupo de pacientes com doença de Chagas em comparação ao grupo controle (p < 0,05 para todas as comparações, Tabela 2 e Figura 1 ). Quando estratificada pelo FRS, a prevalência de ECC > 0 foi maior no grupo controle nos três tercis de escore de risco ( Figura 2 ).

**Tabela 2:** – Comparação da gravidade da doença arterial coronariana (DAC) entre pacientes com doença de Chagas e grupo controle

	Grupos		p*
	Doença de Chagas	Controle	
	Média±DP	Média±DP	
	Mediana (P_**25**_; P_**75**_)	Mediana (P_**25**_; P_**75**_)	
Escore de cálcio coronário	24,7±100,6	49,8± 118,7	0,047
	0 (0; 0)	0 (0; 35)	
Escore de estenose segmentar	0,12± 0,45	0,56±0,80	0,001
	0 (0; 0)	0 (0; 1)	
Número de territórios de perfusão coronária	0,35±0,81	0,77±1,07	0,032
	0 (0; 0)	0 (0; 1)	
Número de segmentos coronários	0,63±1,81	1,35±2,14	0,030
	0 (0; 0)	0 (0; 2)	

DP: desvio padrão; (P_*25:*_ percentil25; P_*75:*_ percentil 75), * teste de Mann-Whitney.

Em relação à presença ou ausência de estenose coronária, 93% dos pacientes com doença de Chagas e 58,1% dos indivíduos do grupo controle não apresentaram nenhuma estenose coronária (p=0,001, Tabela 3 e Figura 1 ).

**Tabela 3:** – Comparação do grau de estenose entre grupo de pacientes com doença de Chagas e grupo controle

Grau de estenose ^*^	Grupos	
	Doença de Chagas’ n (%)	Controle n (%)
Sem estenose	40 (93,0)	25 (58,1)
Estenose leve	1 (2,3)	14 (32,5)
Estenose moderada	2 (4,7)	2 (4,7)
Estenose grave	0 (0)	2 (4,7)

^*^Teste exato de Fisher, p = 0,001.

A frequência da presença de placas coronárias ateroscleróticas foi significativamente mais baixa em pacientes com doença de Chagas que nos sujeitos controles (20,9% versus 41,9%, p = 0,037, Tabela 4 ). Não houve diferença significativa quanto ao tipo de placas coronárias entre os grupos (p=0,237), apesar que 27,9% dos participantes do grupo controle apresentaram placa calcificada versus somente 11,6% do grupo doença de Chagas ( Tabela 4 ). No grupo de pacientes com doença de Chagas, não houve diferença na “carga” da DAC sobre o escore de cálcio entre os grupos, incluindo a ausência de diferença quando comparado à fração de ejeção menor que 55% (grupo cardiomiopatia).

**Tabela 4:** – Caracterização das placas coronárias

Placas ateroscleróticas	Grupos		p
	Chagas’ n (%)	Controle n (%)	
Ausência de placa	34 (79,1)	25 (58,1)	p = 0,037
Presença de placa	9 (20,9)	18 (41,9)	
Tipo de placa#			p = 0,237
Não calcificada	0 (0)	1 (2,3)	
Mista – predominantemente não calcificada	2 (4,7)	3 (7,0)	
Mista	1 (2,3)	0 (0)	
Mista – predominantemente calcificada	1 (2,3)	2 (4,7)	
Calcificada	5 (11,6)	12 (27,9)	

* Teste exato de Fisher. # Análise baseada no paciente– Quando mais de um tipo de placa estava presente, considerou-se o tipo predominante.

Quando a análise foi realizada com base no delineamento caso-controle, a razão de chances ( *odds ratio* , OR) para a presença de calcificação coronária no grupo de pacientes com doença de Chagas foi de 0,27 (IC95%: 0,08 – 0,98, p =0,046). O padrão é similar para ECC > 10 (OR: 0,1; IC95%: 0,13 – 0,78, p=0,028) e para a presença de estenose (OR: 0,06, IC95%: 0,01 – 0,47, p=0,007). Esses resultados mantiveram-se robustos mesmo após ajuste quanto ao FRS, com OR de 0,26 (IC95%: 0,07 – 0,99; p=0,048) para ECC >0; 0,11 (IC95%: 0,01 – 0,87, p=0,04) para ECC >10 e 0,06 (IC95%: 0,01 – 0,47; p=0,001) para a presença de estenose ( Tabela 5 ). Outra análise, utilizando-se o pareamento com escore de propensão, com o método de pareamento de kernel e *bootstrapping* , indicou um efeito médio de -21,6 pontos para o ECC absoluto e -25% para ECC acima de 10 no grupo de pacientes com doença de Chagas comparado ao grupo controle pareado.

**Tabela 5:** – Odds ratio para a presença de qualquer calcificação coronariana, escore de cálcio coronário (ECC > 10), e para a presença de qualquer estenose em pacientes com doença de Chagas

	OR*	IC95%	p
**Não ajustado**			
Presença de qualquer calcificação coronariana	0,27	0,08 – 0,98	0,046
ECC > 10	0,10	0,13 – 0,78	0,028
Presença de estenose	0,06	0,01 – 0,47	0,007
**Ajustado para FRS**			
Presença de qualquer calcificação coronariana	0,26	0,07 – 0,99	0,048
ECC > 10	0,11	0,01 – 0,87	0,04
Presença de estenose	0,06	0,01 – 0,47	0,001

ECC: escore de cálcio coronário; FRS, escore de risco de Framingham. *regressão condicional logística multivariada.

## Discussão

No presente estudo, utilizando técnicas modernas de tomografia computadorizada, nós demonstramos uma prevalência e gravidade mais baixas de DAC em pacientes com doença de Chagas crônica em comparação à população pareada, com perfil de risco para DAC similar. A prevalência de placas coronárias ateroscleróticas em pacientes com infecção crônica por *T. cruzi* foi aproximadamente metade da observada na população controle (não infectada). Importante mencionar que, neste estudo, estudamos a infecção crônica por *T. cruzi* em três estágios diferentes: (1) na fase indeterminada, (2) anormalidade eletrocardiográfica com função do ventrículo esquerdo normal e (3) presença clara de disfunção do ventrículo esquerdo.

### Doença de Chagas e DAC

Estudos prévios investigaram a relação entre doença de Chagas e DAC, com resultados conflitantes. Lopes et al.^23^ examinaram corações submetidos ao exame de necrópsia de 35 pacientes com doença de Chagas e 54 indivíduos não infectados e encontraram que a prevalência de placas coronárias ateroscleróticas (71,4% versus 74,1%, respectivamente) e infarto do miocárdio (8,6% versus 7,4%) foi similar entre os grupos.^23^ Esse resultado contrasta com nossos achados. Acreditamos que a razão para essa aparente discrepância está no fato de que Lopes et al.,^23^ realizaram um estudo patológico baseado em necrópsia e, portanto, é provável que tenham avaliado uma população com doença muito mais avançada. De fato, é possível que alguns desses pacientes com doença de Chagas tenham morrido de complicações da DAC e não por doença de Chagas. Ainda, enquanto Lopes et al.,^23^ estudaram somente indivíduos do sexo masculino, nós incluímos ambos os gêneros no estudo.

Em outro estudo baseado em necropsia, de Morais et al.,^25^ avaliaram 181 corações de pacientes com doença de Chagas e identificaram somente quatro casos de infarto do miocárdio. Um dado interessante foi que todos os casos foram secundários a eventos coronários tromboembólicos, provavelmente de origem em aneurismas apicais do ventrículo esquerdo. Mais importante, o substrato fisiopatológico mais frequentemente associado com infarto do miocárdio, isto é, aterosclerose complicada, não foi encontrado em nenhum paciente desta grande série de autópsias.

Em um estudo observacional prospectivo de nosso grupo, Ianni et al.,^26^ acompanharam 160 pacientes com doença de Chagas crônica, na fase indeterminada por até 14 anos, e conseguiram documentar o desenvolvimento de DAC em apenas dois indivíduos; um com infarto agudo do miocárdio e outro com angina estável. Marin-Neto et al.,^17^ avaliaram 23 indivíduos com doença de Chagas crônica e mostraram anormalidades de perfusão miocárdica detectada por cintilografia com tálio em todos os pacientes. No entanto, a presença de DAC importante não foi observada em nenhum dos 16 pacientes submetidos à angiografia coronária invasiva.

Em outro estudo, Sarabanda et al.,^27^ realizaram angiografia coronária invasiva em 56 indivíduos consecutivos com doença de Chagas e taquicardia ventricular, e demonstraram que a presença de DAC importante não foi observada em nenhum dos pacientes. Mais recentemente, Carvalho et al.,^22^ avaliaram 61 pacientes consecutivos com cardiomiopatia chagásica grave (classe funcional da NYHA III ou IV). Todos os pacientes foram submetidos à angiografia coronária invasiva e a presença de DAC importante (estenose >50%) foi identificada somente em um paciente (1,6%). Esse resultado está de acordo com os nossos, que também demonstraram uma baixa prevalência de DAC importante (4,7%) em pacientes com doença de Chagas crônica.

### ATC versus angiografia invasiva

É importante destacar que existe uma diferença importante entre o presente estudo, em que se utilizou ATC para avaliar presença de DAC, e todos esses estudos prévios em que se usou angiografia coronária invasiva. Apesar do segundo ser considerado o padrão ouro para a avaliação da anatomia coronária e quantificação de estenose coronária, o método consiste em uma luminografia, que não é capaz de detectar ou quantificar placas ateroscleróticas não obstrutivas na parede arterial. Por outro lado, a avaliação do ECC, somada à ATC, não somente é capaz de identificar lesões obstrutivas,^33 - 37^ como também permite a avaliação quantitativa da “carga” aterosclerótica global do indivíduo.^38 - 43^ Assim, trata-se de um instrumento muito mais sensível para a detecção de DAC, particularmente em estágios mais precoces da doença, nas quais a angiografia coronária invasiva pode perder o diagnóstico.

### Limitações e seleção do grupo controle

Nosso estudo possui algumas limitações que devem ser mencionadas. Este é um estudo unicêntrico, com um tamanho amostral relativamente pequeno. O grupo controle foi composto por indivíduos sadios e assintomáticos que se submeteram à ATC somente para estratificação de risco em vez de serem selecionados aleatoriamente da comunidade. Um aspecto importante do estudo refere-se à seleção do grupo controle, que foi uma etapa crítica do estudo. Nós conseguimos selecionar indivíduos comparáveis aos pacientes com doença de Chagas quanto ao risco de desenvolverem DAC, de modo que cada indivíduo do grupo controle foi pareado a um paciente do grupo doença de Chagas quanto ao sexo, idade e FRS. De fato, com exceção da infecção por *T. cruzi* , as características basais de ambos os grupos eram muito similares. A única diferença significativa foi que pacientes com doença de Chagas apresentaram níveis mais elevados de HDL colesterol que indivíduos controles. No entanto, não acreditamos que tal diferença tenha uma influência significativa em nossos resultados, uma vez que, como o grupo controle foi pareado com o grupo de pacientes com doença de Chagas quanto ao FRS, essa pequena diferença nos níveis de HDL foi, ao menos na teoria, compensada pelos outros fatores de risco do escore.^29^ O uso da técnica de pareamento por escore de propensão adicionou mais confiança ao pareamento que realizamos entre os indivíduos com doença de Chagas e controles.

### Implicações clínicas

Infelizmente, no presente estudo, não conseguimos investigar os mecanismos responsáveis pela menor prevalência e gravidade de DAC em pacientes com infecção crônica por T. cruzi. Porém, nossos resultados permitem o levantamento de algumas hipóteses. Uma possibilidade é que, apesar da cuidadosa seleção de um grupo controle pareado, ambos os grupos poderiam ter diferenças genéticas e ambientais que não foram controlados em nosso estudo.

Outra possibilidade fascinante é que a infecção por *T. cruzi* , por si, possa exercer algum efeito protetor contra o desenvolvimento de DAC. Existem evidências preliminares sugerindo que uma enzima derivada do *T. cruzi* , a trans-sialidase, teria o potencial de reduzir a atividade inflamatória e a quantidade de placas ateroscleróticas em modelos experimentais.^28^ É inegável que a mera possibilidade de que essa linha de pesquisa possa resultar no desenvolvimento de uma nova ferramenta terapêutica para a prevenção de DAC é muito excitante.

Por enquanto, a menor prevalência de DAC na doença de Chagas pode sugerir que médicos envolvidos no tratamento de pacientes com doença de Chagas possam utilizar imagens de ATC como uma ferramenta diagnóstica inicial em caso de suspeita de DAC nessa população. Apesar de nossos dados serem insuficiente para embasar uma mudança no tratamento atual, a ATC poderia ser o primeiro passo para uma angiografia coronariana invasiva nesses pacientes, mesmo naqueles em um cenário clínico mais grave, tais como taquicardia e disfunção ventricular.^44^

## Conclusões

No presente estudo, usamos a ATC, uma ferramenta sensível para a detecção de DAC, e demonstramos, de maneira conclusiva, que a DAC é menos prevalente e menos grave em pacientes com doença de Chagas crônica em comparação ao grupo de indivíduos pareados, com risco similar para DAC. Mais estudos são necessários para investigar com mais detalhes os mecanismos responsáveis por esses resultados instigantes.

### Perspectivas

**Competência em conhecimento médico:** A doença de Chagas e a DAC são duas doenças prevalentes na América Latina, e sintomas como dor torácica, podem ser comuns às duas doenças. No entanto, a DAC é menos prevalente em pacientes com doença de Chagas em comparação à população geral com escores de risco de Framingham similares. Isso poderia ajudar médicos durante estratificação de risco de dor torácica.

**Visão translacional** : São necessários estudos futuros para confirmar esses resultados em uma população maior e para identificar possíveis mecanismos envolvidos nesta aparente “proteção” à doença arterial coronariana em pacientes com doença de Chagas.
