# Supplementary material for: Menor Prevalência e Extensão da Aterosclerose Coronária na Doença de Chagas Crônica por Angiotomografia Coronária
Source: Arq Bras Cardiol. 2020 Dec 1;115(6):1051–60. [Article in Portuguese] doi: 10.36660/abc.20200342 (PMC8133709; doi:10.36660/abc.20200342)
Supplement: Translation

# Translation: Lower Prevalence and Severity of Coronary Atherosclerosis in Chronic Chagas’ Disease by Coronary Computed Tomography Angiography

**Authors:** Savio Cardoso, Clerio Francisco de Azevedo, Fábio Fernandes, Barbara Ianni, Jorge Andion Torreão, Mateus Diniz Marques, Luiz Francisco Rodrigues de Ávila, Raul Santos, Charles Mady, Roberto Kalil-Filho, José Antônio Franchine Ramires, Marcio Sommer Bittencourt, Carlos E. Rochitte

**Affiliations:** 1 Brazil Universidade de São Paulo Faculdade de Medicina Hospital das Clínicas Instituto do Coração, São Paulo, SP - Brazil; 2 USA Duke University Hospital - Medicine/Cardiology, North Carolina - USA; 3 Brazil Hospital Universitário de Santa Maria, Santa Maria, RS - Brazil; 4 Brazil Hospital Sírio-Libanês, São Paulo, SP - Brazil; 5 Brazil Hospital Israelita Albert Einstein, São Paulo, SP - Brazil; 6 Brazil Universidade de São Paulo Hospital Universitário de São Paulo, São Paulo, SP - Brazil; 7 Brazil Hospital do Coração (HCOR) Associação Beneficente Síria, São Paulo, SP - Brazil

**Keywords:** Chagas Disease/physiopathology, Atherosclerosis, Coronary Artery Disease, Tomography, Computerized/methods, Score Calcium

## Abstract

**Background:**

In Chagas’ disease endemic regions, there has been for many years a recurrent empirical observation that coronary artery disease (CAD) is uncommon in patients with Chagas’ disease. Previous pathological and invasive coronary angiography studies led to controversial results.

**Objective:**

We sought to investigate whether CAD is less prevalent and less severe in patients with chronic Chagas’ disease when compared with a matched population with a similar CAD risk profile.

**Methods:**

A total of 86 participants, 43 consecutive patients with chronic Chagas’ disease and 43 asymptomatic individuals, without any prior history of cardiac disease or known CAD (control group), were included. Patients and controls were matched according to gender, age, and Framingham risk score. All participants underwent coronary calcium scoring and coronary computed tomography angiography on a 320-row detector scanner. Statistical significance level adopted was p < 0.05.

**Results:**

The coronary artery calcium score (CACS) was significantly lower in patients with Chagas’ disease than in controls (p<0.05). The presence of coronary atherosclerotic plaques was significantly less frequent in patients with Chagas’ disease than in controls (20.9% versus 41.9%, p=0.037). After adjustment for the Framingham score, the odds ratio for the presence of any coronary artery calcium (CAC) in Chagas patients was 0.26 (95%CI: 0.07-0.99, p=0.048). The pattern is similar for CACS > 10 (OR: 0.11, 95%CI: 0.01-0.87, p=0.04) and for the presence of any stenosis (OR: 0.06, 95%CI: 0.01-0.47, p=0.001). Propensity score matching also indicated an effect of Chagas disease on the CACS (-21.6 points in the absolute score and 25% less of patients with CACS >10, p=0.015).

**Conclusions:**

CAD is less prevalent and less severe in patients with chronic Chagas’ disease when compared with a matched population with a similar CAD risk profile. (Arq Bras Cardiol. 2020; 115(6):1051-1060)

## Introduction

Approximately 20,000 people die annually from Chagas’ disease and 100 million people are at risk of contracting the infection worldwide. It is endemic throughout much of Mexico, Central America, and South America, where as many as 6 million people are infected.^1 , 2^In the United States, an estimated 300,000 individuals are infected with *Trypanosoma cruzi* ( *T. cruzi)* , most of who acquired the infection in endemic areas and then migrated to North America.^3 - 5^

Chagas’ cardiomyopathy is essentially a myocarditis. The inflammatory process, although more intense in the acute phase, is clinically silent but continuous in patients with chronic infection.^6 , 7^After the acute illness, patients enter the indeterminate phase, which is asymptomatic and may last indefinitely. About 20 to 30% of people with chronic *T. cruzi* infection eventually develop clinical disease, predominantly cardiac. Cardiac disease usually begins with conduction abnormalities such as right bundle branch block and/or left anterior fascicular block, which may be followed by dilated cardiomyopathy years later.^8^Heart failure and other associated abnormalities,^9 - 14^including sympathetic autonomic dysfunction^15^can be seen at this phase. In later stages, apical aneurysms and thrombus formation can be frequent findings.

Importantly, during the chronic cardiomyopathy phase, a significant proportion of patients complain of atypical chest pain. Yet, the prevalence of coronary artery disease (CAD) in these patients has been found to be consistently low. Therefore, expert investigators in Chagas’ disease have raised the hypothesis that patients with Chagas’ disease would have less CAD than individuals without Chagas’ disease but with an otherwise similar CAD risk profile. Still, the relationship between Chagas’ disease and coronary atherosclerotic disease remains controversial.^16^It has been demonstrated that abnormal myocardial perfusion and even extensive myocardial scarring may occur in patients with Chagas’ disease in the absence of epicardial coronary stenosis.^17 , 18^Myocardial fibrosis in Chagas’ disease patients can be detected in early phases of the disease and precisely quantified by cardiac magnetic resonance;^19 , 20^its magnitude has proven prognostic significance.^21^

Despite the high prevalence of CAD risk factors, including smoking, initial reports indicating a very low prevalence of significant CAD by invasive coronary angiography in patients with severe Chagas cardiomyopathy have raised the suspicion of a distinct prevalence of CAD in Chagas patients.^22 , 23^The influence of Chagas disease on CAD and other chronic degenerative diseases including neoplastic diseases^24^has biological plausibility based on the presence of common mechanistic factors such as chronic inflammation, fibrosis, increase in free radical and low-density lipoprotein levels and decrease in nitric oxide levels. Nonetheless, studies that described the prevalence of CAD in patients with Chagas’ disease have reported conflicting results. While some studies have found a prevalence that is similar to that observed in the general population,^23^several studies have suggested that the prevalence of CAD in patients with Chagas’ disease could be lower than that of a non-infected population with similar clinical risk scores for CAD.^17 , 22 , 25 - 27^There is also some preliminary basic evidence that *T. cruzi* infection itself might exert a protective effect against the development of CAD in chronically infected subjects.^28^This would be possible by the potential action of trans-sialidase produced by T. cruzi in reducing mycoplasma infection, which could be involved in inflammation associated with CAD.^28^

In the present study, we used state-of-the-art coronary computed tomographic techniques to determine whether patients with chronic Chagas’ disease have a lower prevalence and/or severity of CAD when compared with a matched population of asymptomatic and with no previous history of CAD, but with a similar CAD risk profile.

## Methods

### Population

A total of 43 consecutive patients with chronic Chagas’ disease were prospectively recruited from our specialized outpatient clinic. All patients were serologically positive for Chagas’ disease by at least two different techniques: ELISA and indirect immunofluorescence. They were classified in three subgroups: (1) patients in the indeterminate phase, (2) patients with electrocardiographic abnormalities, and (3) patients with heart failure. The indeterminate subgroup (15 patients, 34.9%) was defined as asymptomatic patients with chronic Chagas’ disease but without abnormalities on chest X-ray, electrocardiogram (ECG), echocardiography or contrasted X-ray studies of esophagus and colon. The ECG subgroup (12 patients, 27.9%) was defined as patients with electrocardiographic abnormalities, but no abnormalities on global or segmental left ventricular (LV) systolic function and with LV ejection fraction ≥ 55% by echocardiography. Patients with LV ejection fraction < 55% were assigned to the cardiomyopathy subgroup (16 patients, 37.2%).

Additionally, a total of 43 asymptomatic individuals, without any prior history of cardiac disease or CAD, and no family history of early CAD, that underwent coronary computed tomography angiography (CTA) for risk stratification only were included as control group. These 43 patients were selected from a larger control group of 124 consecutive individuals that filled the matching criteria for each Chagas disease patient. A second individual who filled the matching criteria was not included and allocated to the backup control group. For selection of the control group, three levels of matching were used for each of the Chagas’ disease patients (case-control 1x1): first, a Framingham risk score (FRS) within the same risk strata (low, intermediate and high);^29^second, age differences < 5 years; and third, the same gender. Also, since there were no patients with diabetes mellitus in the Chagas’ disease group, we also excluded diabetic patients from the control group. The FRS was calculated using the model described by D’Agostino et al.,^16^and was based on the following clinical parameters: age, total and high-density lipoprotein cholesterol, systolic blood pressure, treatment for hypertension, smoking and diabetes status. All 43 individuals underwent serological testing to exclude Chagas’ disease.

All patients signed an informed consent form approved by our local institutional ethics committee. In both groups we excluded patients with renal insufficiency, defined as serum creatinine > 1.5mg/dL and/or creatinine clearance of <60mL/min/1.72m^2^, by the MDRD formula (Modification of Diet for Renal Disease).

### Coronary computed tomographic angiography (Coronary CTA)

All patients underwent coronary CTA on a 320-detector row scanner ( *AquillionOne* ^TM^ *– Canon Medical Systems Corporation,* Otawara, Japan) after fasting for at least 4 hours. Prior to the exam, the patients answered a questionnaire on cardiovascular risk and were examined for heart rate (HR) and blood pressure.

Acquisition protocol included the coronary artery calcium score (CACS) and coronary CTA. CACS was determined using a tube rotation time of 370ms, tube voltage of 120 kV, current of 300mA, 320x0.5-mm collimation with 3-mm slice thickness reconstructed images obtained per heartbeat during diastole.

For coronary CTA, participants with heart rate over 65 bpm received up to 20mg of intravenous metoprolol. All participants received sublingual nitrates if their systolic blood pressure were greater than 90 mmHg. Superior and inferior limits for acquisition were defined on the CACS imaging. A real time tracking of contrast bolus propagation ( *Sure Start* ^TM^– *Toshiba Medical Systems Corporation,* Otawara, Japan) was used to detect steep increase in signal intensity on the descending aorta. The acquisition was started at a threshold of 150 HU. Non-ionic iodinated contrast (70mL, with iodine concentration of 370mg/mL) (Iopamiron 370 – Schering, São Paulo, Brazil, under license of BRACCO - Italy) was then administered by a power injector ( *Stellant* ^TM^– *Medrad,* Indianola, PA, USA) at a 5mL/s rate followed by 40mL of saline chaser.

Coronary CTA image acquisition occurred within one single heart beat (HR less than 65 bpm) according to a prospectively ECG-triggered protocol and during inspiratory breath-hold.^30 , 31^Coronary CTA acquisition parameters depended on participants’ body mass index (BMI): up to 23 kg/m^2^, tube voltage of 100kV and tube current of 450-550mA; BMI from 24 to 39 kg/m2, 120kV and 400-580mA, respectively; BMI greater than 40kg/m^2^, 135kV and 510mA, respectively. Collimation depended on longitudinal size of the heart and varied from 120 to 160mm, with 0.5mm thick slice acquisitions (240x0.5mm to 320x0.5mm). Typical radiation dose for this protocol is below 10 mSv.

### Coronary CTA Analysis

CACS analysis used standard protocol described by Agatston et al.,^32^using at least 3 pixels equal to or greater than 130 HU to define calcium.

Coronary CTA images were reconstructed immediately after scan completion in a consistent manner in order to identify motion-free coronary artery images. ECG-gated datasets were reconstructed at 75% of the cardiac cycle. In case of insufficient image quality, additional phases were reconstructed at 5% increments. Multiple phases were used for image interpretation if the phase of minimum coronary artery motion was different for different arteries. All images were analyzed in a *Vitrea* ^TM^ *FX* workstation ( *Vital Images Inc,* Plymouth, MN, EUA) by two experienced coronary CTA who were unaware of any clinical information. The observers could use all tools available in the workstation to analyze the images, such as axial views, multiplanar and curved reformatting, maximal intensity projection and 3D volume rendering. For the discrepancies, a consensus between the two readers was reached for the presence and type of atherosclerotic plaque and the degree of stenosis per each coronary segment. For coronary segmentation we used a 19-segment model, previously described in the CorE-64 trial.^33^

CAD was defined as the presence of any coronary atherosclerotic plaque, even in absence of luminal obstruction. Significant obstructive CAD was defined as luminal reduction equal to or greater than 50% of the reference luminal diameter (segment immediately distal without evident disease). Atherosclerotic plaque was classified by a qualitative analysis as: A – non-calcified plaque, B – predominantly non-calcified plaque, C – mixed plaque, D – predominantly calcified plaque and E – calcified plaque. When more than one type of plaque was present, the most predominant type in each patient was considered for analysis. The degree of coronary stenosis was visually classified by the two observers according to the segment stenosis score: 0 – absence of luminal reduction, 1- mild stenosis (< 50%), 2- moderate stenosis (50-69%) and 3 – severe stenosis (≥ 70%). Figure 1 shows two examples of Chagas’ disease patients with and without CAD.

**Figure 1:** – Coronary computed tomography angiography (coronary CTA) images of case examples of Chagas’ patients without (top row) and with (bottom row) coronary artery disease (CAD). Panels A, B and C show three different views of the left anterior descending artery (LAD) of both patients. On the bottom row, red arrows indicate partially calcified plaque at proximal LAD without stenosis (predominantly cal mixed plaque), and yellow arrows indicate a non-calcified plaque with significant luminal stenosis.

### Statistical Analysis

All continuous variables are presented as mean ± standard deviation, and all categorical variables are reported as percentage or absolute number. Continuous variables with normal distribution were described using the mean and standard deviation and continuous variables without normal distribution were described using the median and interquartile range. Shapiro-Wilk test was used to check for normal distribution. For comparison between groups, we used the paired Student’s *t* test for variables with normal distribution, and the Mann-Whitney test for non-parametric variables. For comparisons of categorical variables, we used Pearsons’ chi-square test or Fisher’s exact test as appropriate. For the multivariable analysis we performed conditional logistic regression analysis adjusted for the FRS for each of the binary outcomes of CACS equal to or greater than zero; CACS lower than 10 or greater or equal to 10 and presence of coronary obstruction (defined as >50% stenosis on visual assessment). Propensity score matching, using kernel method and bootstrapping was used for additional matching between Chagas’ disease and control patients. There are no data in the literature on the prevalence of atherosclerotic coronary plaque in Chagas disease patients measured by computed tomography (CT), therefore we used an exploratory or convenience sample size. The study was designed to have a matched control group, so 43 Chagas patients had to be matched to 43 normal controls with similar sex, age range and FRS. Analyses were performed with Stata software, version 13.0 (StataCorp, College Station, Texas). All tests were two-tailed, and a value of p<0.05 was considered indicative of statistical significance.

## Results

The clinical characteristics of all participants are shown in Table 1 . In Chagas’ disease group, 27 women were (62.8%) and the mean age was 54.2 ± 8.3 years, and in the control group, there were also 27 women (62.8%), and the mean age was 55.0 ± 7.1 years. Gender, age, and the FRS were similar, highlighting the effective matching of both groups. The Chagas’ disease group was found to have higher levels of HDL cholesterol than the control group (p=0.030, Table 1 ). No patient was excluded due to limited coronary CTA image quality, and no adverse event related to the procedures of this study was reported.

**Table 1:** – Clinical characteristics

Characteristics	Groups		p
	Chagas’ (n=43)	Control (n=43)	
Age (years)	54.18 ± 8.26	55.00 ± 7.12	0.626*
Male	16 (37.2%)	16 (37.2%)	1.000**
BMI	27.4±4.2	28.9±4.8	0.127*
Coronary CTA HR (bpm)	63.4±5.1	62.2±3.7	0.215*
HDL cholesterol (mg/dL)	51.0 ± 12.4	45.9 ± 9.2	0.030*
Total cholesterol (mg/dL)	201.5 ± 36.3	224.3 ± 84.6	0.108*
Treated hypertension	20 (46.5%)	24 (55.8%)	0.388**
Current smoker	04 (9.3%)	04 (9.3%)	1.000**
Framingham risk score	3 (1; 8)^†^	4 (1; 8)^†^	0.682***

BMI: body mass index; CTA: computed Tomography Angiography; HR: heart rate; bpm: beats per minute; HDL: high density lipoprotein. †: Median (P25; P75); *: Student t test; **: Pearsons’ chi-square test; ***: Mann-Whitney test.

The CACS was significantly lower in patients with Chagas’ disease than in controls. Similarly, the severity of coronary stenosis, number of coronary territories and number of coronary segments with CAD were significantly lower on Chagas’ disease group compared to control group (p < 0.05 for all comparisons, Table 2 and Figure 1 ). When stratified by the FRS the prevalence of CAC >0 is higher in the control group across the three FRS tertiles ( Figure 2 ).

**Table 2:** – Comparison of coronary artery disease (CAD) severity between patients with Chagas’ disease and control group

	Groups		p*
	Chagas’ disease	Control	
	Mean±SD	Mean±SD	
	Median (P_**25**_; P_**75**_)	Median (P_**25**_; P_**75**_)	
Coronary calcium score	24.7±100.6	49.8± 118.7	0.047
	0 (0; 0)	0 (0; 35)	
Segment stenosis Score	0.12± 0.45	0.56±0.80	0.001
	0 (0; 0)	0 (0; 1)	
Number of coronary territories	0.35±0.81	0.77±1.07	0.032
	0 (0; 0)	0 (0; 1)	
Number of coronary segments	0.63±1.81	1.35±2.14	0.030
	0 (0; 0)	0 (0; 2)	

SD: standard deviation; (P25: 25th percentile; P75: 75th percentile), * Mann-Whitney test.

**Figure 2:** – Relationship between Chagas’ disease and the prevalence of CAD. A. Prevalence of any coronary artery calcium (coronary artery calcium score, CACS > 0) in patients with Chagas’ disease and in controls stratified by the Framingham Risk Score (FRS) tertiles. B. Prevalence of CACS > 10 in patients with Chagas’ disease and in controls stratified by Framingham Risk Score tertiles.

Regarding the presence versus absence of coronary stenosis, 93% of Chagas’ disease patients and 58.1% of control group participants did not have any coronary stenosis (p = 0.001, Table 3 and Figure 1 ).

**Table 3:** – Comparison of the degree of coronary stenosis between Chagas disease and control groups

Degree of stenosis^*^	Groups	
	Chagas’ n (%)	Control n (%)
No stenosis	40 (93.0)	25 (58.1)
Mild stenosis	1 (2.3)	14 (32.5)
Moderate stenosis	2 (4.7)	2 (4.7)
Severe stenosis	0 (0)	2 (4.7)

^*^Fisher’s exact test, p = 0.001.

The presence of coronary atherosclerotic plaques was significantly less frequent in patients from the Chagas’ disease group than in subjects from the control group (20.9% versus 41.9%, p = 0.037, Table 4 ). No significant differences were found in the type of coronary plaques between the groups (p=0.237), although 27.9% of participants of the control group had calcified plaque versus only 11.6% in the Chagas’ disease group ( Table 4 ). Within the Chagas disease group, there were no differences in CAD burden based on calcium score among groups, including no differences compared to those with ejection fraction less than 55% (cardiomyopathy group).

**Table 4:** – Characterization of coronary plaques

Atherosclerotic plaques	Groups		p
	Chagas’ disease n (%)	Control n (%)	
No plaque	34 (79.1)	25 (58.1)	p = 0.037
Plaque present	9 (20.9)	18 (41.9)	
Type of plaque#			p = 0.237
Non calcified	0 (0)	1 (2.3)	
Mixed – predominat non calcified	2 (4.7)	3 (7.0)	
Mixed	1 (2.3)	0 (0)	
Mixed – predominant calcified	1 (2.3)	2 (4.7)	
Calcified	5 (11.6)	12 (27.9)	

* Fisher’s exact test. # Patient-based analysis – the most predominant type of plaque was assigned

When the analysis was performed based on the case-control design, the odds ratio for the presence of any CAC in Chagas’ disease patients was 0.27 (95%CI: 0.08 – 0.98, p =0.046). The pattern is similar for CACS > 10 (OR: 0.1, 95%CI: 0.13 – 0.78, p=0.028) and for the presence of any stenosis (OR: 0.06, 95%CI: 0.01 – 0.47, p=0.007). These results remained robust even after adjustment for the FRS, with OR of 0.26 (95%CI: 0.07 – 0.99, p=0.048) for CACS>0, 0.11 (95%CI: 0.01 – 0.87, p=0.04) for CACS>10 and 0.06 (95%CI: 0.01 – 0.47, p=0.001) for the presence of any stenosis ( Table 5 ). An additional analysis using propensity score matching, with kernel matching method and bootstrapping, indicated an average effect of – 21.6 points for the absolute CACS and -25% for CACS above 10 in Chagas disease patients when compared to the matched control group.

**Table 5:** – Odds ratio for the presence of any coronary artery calcium, coronary artery calcium score (CACS > 10), and for the presence of any stenosis in patients with Chagas disease

	OR*	95% CI	p
**Unadjusted**			
Presence of any CAC	0.27	0.08 – 0.98	0.046
CACS > 10	0.10	0.13 – 0.78	0.028
Presence of any stenosis	0.06	0.01 – 0.47	0.007
**Adjusted for FRS**			
Presence of any CAC	0.26	0.07 – 0.99	0.048
CACS > 10	0.11	0.01 – 0.87	0.04
Presence of any stenosis	0.06	0.01 – 0.47	0.001

CAC: coronary artery calcium score; FRS:Framingham risk score. *Multivarate logistic conditional regression.

## Discussion

In the present study, using state-of-the-art computed tomographic techniques, we demonstrated a lower prevalence and severity of CAD in chronic Chagas’ disease patients when compared with a matched population with a similar CAD risk profile. The prevalence of coronary atherosclerotic plaques in patients with chronic *T cruzi* infection was approximately half of that observed in the control non-infected population. Importantly, in the present study, we studied chronic *T. cruzi* infection in three different stages: (1) the indeterminate phase, (2) ECG abnormality with normal LV function and (3) overt LV dysfunction.

### Chagas’ disease and CAD

Previous studies have investigated the relationship between Chagas’ disease and CAD, and the results have been conflicting. Lopes et al.^23^examined autopsied hearts of 35 chronic Chagas’ disease patients and 54 non-infected individuals and found that the prevalence of coronary atherosclerotic plaques (71.4% versus 74.1%, respectively) and myocardial infarction (8.6% versus 7.4%, respectively) was similar in both groups.^23^This is in contrast with our results. We believe the reason for this apparent discrepancy relies on the fact that Lopes et al.^23^performed an autopsy-based pathology study and, therefore, it is likely that they evaluated a population with much more advanced disease. In fact, it is possible that some of the patients in the Chagas’ disease group died of CAD complications and not Chagas’ disease. Moreover, unlike Lopes et al.^23^who studied male individuals only, we included both genders in the present study.

In another autopsy-based study, de Morais et al.^25^examined 181 hearts of chronic Chagas’ disease patients and identified only four cases of myocardial infarction. Interestingly, all cases were secondary to thromboembolic coronary events, probably originated in apical LV aneurysms. Most importantly, the pathophysiological substrate most frequently associated with myocardial infarction, i.e. complicated atherosclerosis was not observed in any patient of this large autopsy series.

In a prospective observational study from our group, Ianni et al.^26^followed 160 patients in the indeterminate phase of chronic Chagas’ disease for up to 14 years and were able to document the development of CAD in only two individuals; one presented an acute myocardial infarction and the other stable angina. Marin-Neto et al.^17^evaluated 23 subjects with chronic Chagas’ disease and demonstrated that myocardial perfusion abnormalities as detected by thallium scintigraphy were present in all patients. Nevertheless, the presence of significant CAD was not observed in any of the 16 patients that underwent invasive coronary angiography.

In another study, Sarabanda et al.^27^performed invasive coronary angiography in 56 consecutive subjects with chronic Chagas’ disease and ventricular tachycardia and demonstrated that the presence of significant CAD was not observed in any of those patients. More recently, Carvalho et al.^22^evaluated 61 consecutive patients with severe Chagas’ cardiomyopathy (NYHA functional class III or IV). All patients underwent invasive coronary angiography, and the presence of significant CAD (> 50% stenosis) was identified in only one patient (1.6%). These finding are in agreement with our results, which also demonstrated a low prevalence of significant CAD (4.7%) in chronic Chagas’ disease patients.

### Coronary CTA versus invasive angiography

It is important to highlight that there is an important difference between the present study, that used coronary CTA to evaluate the presence of DAC, and all these previous reports that used invasive coronary angiography. Even though the latter is considered the gold standard for the assessment of coronary anatomy and quantification of coronary stenosis, it consists of a luminography, i.e., it is not able to detect or quantify the amount of non-obstructive atherosclerotic plaques in the arterial wall. In contrast, coronary calcium scoring plus coronary CTA is not only capable of identifying obstructive lesions,^33 - 37^but also allows for the quantitative assessment of the global atherosclerotic burden of the individual.^38 - 43^Therefore, it is a much more sensitive tool for the detection of CAD, particularly in the earlier stages of the disease, in which the invasive coronary angiography might miss the diagnosis.

### Limitations and selection of the control group

Our study has some limitations that must be recognized. This is a single-center study with a relatively small sample size. The control group was composed of healthy and asymptomatic individuals that underwent coronary CTA for risk-stratification only and not randomly selected from the community. One important aspect of the present study relates to the selection of the control group, which was a critical step. We were able to select individuals comparable to Chagas’ disease patients in the risk to develop CAD, so that each individual of the control group was matched to one patient in the Chagas’ disease group for gender, age and the FRS. Indeed, except for the *T. cruzi* infection, the baseline characteristics of both groups were very similar. The only significant difference was that patients in the Chagas’ disease group demonstrated higher levels of HDL cholesterol than the individuals in the control group. However, we do not believe this difference had a significant influence on our results, particularly because, since the control group was matched regarding the FRS, this small difference in HDL cholesterol level was, at least theoretically, counterbalanced by the other risk factors of the score.^29^The use of the propensity score matching technique added an extra layer of confidence in our matching between Chagas’ disease and control group.

### Clinical implications

Unfortunately, in the present study, we were not able to investigate the underlying mechanisms of the lower prevalence and severity of CAD in individuals with chronic *T. cruzi* infection. Nevertheless, our results allow us to raise some hypotheses. One possibility is that, despite the careful selection of a matched control group, both populations could have genetic or environmental differences that were not controlled in our study.

Another fascinating possibility is that the *T. cruzi* infection itself could exert some protective effect against the development of CAD. There is some preliminary evidence suggesting that an enzyme derived from the *T. cruzi,* called trans-sialidase, could have the potential to reduce the inflammatory activity and the amount of atherosclerotic plaques in experimental models.^28^It is undeniable that the mere possibility that this line of research could result in the development of a novel therapeutic tool for the prevention of CAD is very exciting.

In the meanwhile, the lower prevalence of CAD in Chagas’ disease may suggest that physicians caring for Chagas’ disease patients could utilize coronary CTA imaging as a first step diagnostic tool for suspected CAD in this population. Although our data is still not enough to support a change in current patient management, CTA could potentially be a gatekeeper for invasive coronary angiography in these patients, even those with more severe clinical scenario, such as ventricular tachycardia and dysfunction.^44^

## Conclusions

In the present study, we used coronary CTA, which is a sensitive tool for the detection of CAD, and conclusively demonstrated that CAD is less prevalent and less severe in patients with chronic Chagas’ disease when compared with a matched population with a similar CAD risk profile. Future studies will be necessary to investigate in greater detail the underlying mechanisms of these instigating findings.

### Perspectives

**Competency in medical knowledge:** Chagas’ disease and CAD are two prevalent diseases in Latin America, and symptoms such as chest pain, might be similar in patients with either of these diseases. Nonetheless, CAD is less prevalent in patients with Chagas’ disease compared to general population with similar Framingham risk scores. This could help clinicians during risk stratification of chest pain.

**Translational outlook:** Future research is needed to confirm these findings in a large population and to identify potential mechanisms involved in this apparent “protection” for CAD in patients with Chagas’ disease.
